# Hands and feet radiologic involvements in systemic sclerosis

**DOI:** 10.1186/s41927-023-00336-9

**Published:** 2023-05-20

**Authors:** Fatemeh Badiee, Alireza Fatemi, Reza Zahedpasha, Mohammad Hadi Gharib, Mohammadhassan Jokar, Somayeh Livani, Mehrdad Aghaie, Nafiseh Abdolahi

**Affiliations:** 1 Golestan Rheumatology Research Center, Golestan University of Medical Sciences, Gorgan, Iran; 2 Student Research Committee, Golestan University of Medical Sciences, Gorgan, Iran; 3 Department of Radiology, School of Medicine, 5Th Azar Hospital, Gorgan, Golestan Iran; 4 Golestan University of Medical Sciences, Gorgan, Golestan Iran, Islamic Republic of; 5 Clinical Research Development Unit (CRDU), Sayad Shirazi Hospital, Gorgan, Iran, Islamic Republic of

**Keywords:** Systemic Sclerosis, Arthropathy, Acro-osteolysis, Hand Stiffness, Joint space narrowing, Juxta-articular osteoporosis

## Abstract

**Aim:**

Systemic sclerosis (SSc) is a rare autoimmune disorder characterized by vascular and fibrosing involvement of the skin and internal organs. In this study, we determined the prevalence and characteristics of radiological hands and feet involvements in Iranian SSc patients to identify the associations between clinical features and radiologic findings.

**Methods:**

43 SSc patients (41 women and 2 men), with a median age of 44.8 years (ranges 26–70 years) and a mean disease duration of 11.8 years (ranges 2–28 years) were studied in this cross-sectional study**.**

**Results:**

42 patients had radiological changes both in their hands and feet. Only one patient had alteration just in hand. The most frequent changes that we found in hand were Juxta-articular Osteoporosis (93%), Acro-osteolysis (58.2%), and Joint Space Narrowing (55.8%). The prevalence of joint space narrowing or acro-osteolysis was higher in subjects with active skin involvement [modified Rodnan skin score (mRSS) > 14] [16/21 vs. 4/16 for patients with inactive skin involvement (mRSS < 14); *p* = 0.002]. The most frequent changes that we found in the foot were Juxta-articular Osteoporosis (93%), Acro-osteolysis (46.5%), Joint Space Narrowing (58.1%), and subluxation (44.2%). The presence of anti-ccp antibody was detected in 4 (9.3%), while positive rheumatoid factor was found in 13 (30.2%) of SSc patients.

**Conclusion:**

This study corroborates that arthropathy is common in SSc patients. The introduction of the specific radiological involvements of SSc needs to be confirmed by further studies, in order to define the appropriate prognosis and treatment of patients.

## Introduction

Systemic sclerosis (SSc) is a rare autoimmune disorder characterized by a vascular and fibrosing involvement of the skin and internal organs [1]. Hand stiffness and articular pain are two of the highest rated symptoms in terms of frequency and moderate to severe impacting the quality of life of patients with SSc [2]. To date many distinct radiographic abnormalities of hand and foot have been recognized. Different proposals of radiographic classification patterns have been published [3, 4]. But there is not any specific pattern of radiological involvement recognized in SSc. There is a critical need for the development and validation of some uniform system for identification of joint involvements in SSc (ie, as Sharp as findings known for rheumatoid arthritis). While the frequency of some findings such as foot subluxation in past studies were close and ranging from 13.1 [3] to 16% [5]; there were findings like hand joint space narrowing with controversial reported frequencies, ranging from 17.9% [6] to 75.3 % [7]. Most reports on the prevalence of radiological involvements in SSc were on hand and from Western countries (Europe [3–5, 7–17], Canada [18], United States [19, 20] and Africa [21] with only four previous reports from Asia [6, 22–24]. Few studies evaluated radiologic changes in the feet and they were from Europe [3, 5, 25], Africa [21] and only one old from Asia [26]. Increasing our understanding of hand and foot involvements may make it possible to improve therapeutic approaches. Keeping in mind these findings, this study was undertaken first to determine the prevalence and characteristics of radiological hand and foot involvements in Iranian SSc patients and second, to identify disease–phenotype associations. To better investigation of radiological findings, we included only patients with disease duration (measured from the onset of the first symptom) ≥ 2 years and excluded those with rheumatoid arthritis-systemic sclerosis overlap syndromes or other overlap syndromes.

## Methods

All SSc patients, fulfilling the American College of Rheumatology classification criteria for SSc [27], referred to Sayyad Shirazi Medical Education Center and rheumatologists’ offices in Gorgan (Golestan, Iran), were invited by telephone call. SSc patients with disease duration < 2 years or overlap syndromes were excluded. A total of 43 patients were evaluated in this cross-sectional study. All patients gave informed consent for all procedures and underwent skin examination. Active skin involvement was determined on the basis of modified Rodnan skin score (mRSS > 14) [28]. The following laboratory tests; fasting blood glucose (FBS), Hemoglobin A1c (HbA1c), rheumatoid factor (RF) and anti-cyclic citrullinated peptide (Anti-CCP) antibody were carried out. Radiographic examination, obtained at the moment of laboratory evaluation and clinical data collection. Standard anteroposterior views of both hands and feet were obtained for all patients. All radiographs were evaluated by a radiologist using a predefined set of radiographic findings according to the previous studies [3, 7]. Radiographs were examined directly on computer, and the two radiologists was blinded to serological data and severity of the SSc patients` disease. Each finding was separately scored [0 (normal), 1 (mild), 2 (moderate), or 3 (severe)]. For the classification of extent of degenerative changes in X-ray, there is a well-known classification system called Kellgren Lawrence classification, which is a 4-class category. Class 2, 3 and 4 are assumed as mild, moderate and severe degenerative changes, respectively [29]. The clinical data of age, sex, cutaneous subtype as defined by Leroy et al. [30], disease duration (measured from the onset of the first symptom) and heart involvement; were collected from patients’ records. Occurrence of pericarditis, left ventricular ejection fraction (LEVF) < 55%, pericardial effusion, valve regurgitation, chamber hypertrophy, and primary pulmonary arterial pressure (PAP) on echocardiography were considered signs of scleroderma heart involvement. Pulmonary involvement was assessed by a new chest radiograph, reduced forced vital capacity (FVC) in pulmonary function tests (PFT), and fibrosis in high-resolution computed tomography (HRCT) of patients’ records. Pulmonary arterial hypertension (PAH) was defined as PAP higher than 25 mmHg on echocardiography. Gastrointestinal involvement was defined from the simultaneous study of these patients; based on the questionnaire about gastrointestinal symptoms [University of California at Los Angeles Scleroderma Clinical Trial Consortium (UCLA SCTS) 2.0] [31] and the abnormal findings of barium swallow and computed tomography (CT) enterography. Statistical analysis was undertaken using statistical packages for social sciences (16th version). Percentage, mean and standard deviation were used to describe the data, and Chi-square test was used to examine the relationship between variables. Also, *P* values less than 0.05 were considered statistically significant.

## Results

**Fig. 1:**
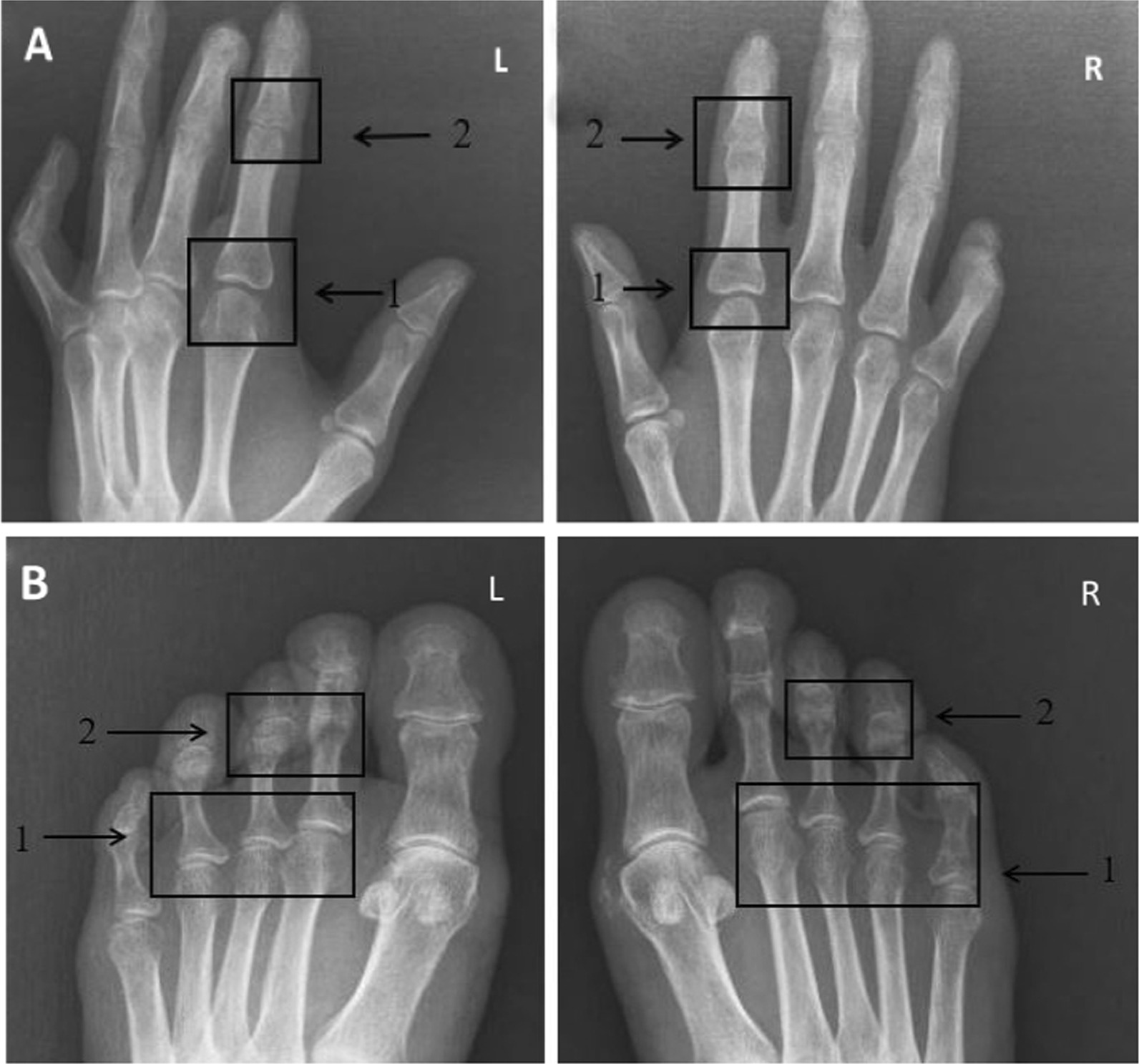
**A** Hands radiographic images. **B** Foot radiographic images. A 46-year-old woman with SSc presented with joint pain. Hands and foot radiographic images show the following abnormality (arrows) on the left and right: 1-Bone erosion representing Juxta-articular Osteoporosis 2-Joint Space Narrowing with an abnormal distance between bones

We studied 43 SSc patients (41 women and 2 men), with a median age of 44.8 years (ranges 26–70 years) and a mean disease duration of 11.8 years (ranges 2–28 years). 23 patients had lSSc and 18 patients had dSSc (Two patients` disease type was unspecified). Other detailed clinical and laboratory data are provided in Tables 1 and 4. 2 patients had radiological changes both in their hands and feet. Only one patient had modifications just in hand. She was middle-aged and had a long disease duration, diffuse subset, inactive skin involvement, and GI involvement. In particular, severe radiographic findings were less prevalent in the feet than in the hands (18.6% vs. 39.5%; *P* value < 0.05). The most frequent changes that we found in hand were Juxta-articular Osteoporosis (93%), Acro-osteolysis (58.2%), and Joint Space Narrowing (55.8%). Table 2 shows in detail the prevalence and distribution of each radiological change observed in hand. Figure 1A. The most frequent changes that we found in the foot were Juxta-articular Osteoporosis (93%), Acro-osteolysis (46.5%), Joint Space Narrowing (58.1%), and subluxation (44.2%). Foot radiological findings of patients with SSc are detailed in Table 3 and Fig. 1B.

**Table 1:** Clinical features of the SSc patients (43 patients)

Sex (F/M)	41/2
Age (years)	44.8 ± 12.5
Young (< 44 years)	21 (48.8%)
Middle aged (> 44 years)	22 (51.2%)
Disease duration (years)	11.8 ± 7.7
Short (< 10 years)	24 (55.8%)
Long (> 10 years)	19 (44.2%)
*Subset (number; %)*	
LcSSc*	23 (56.1%)
DcSSc**	18 (43.9%)
*Autoantibody profile: (number; %)*	
RF positive	13 (30.2%)
Anti-CCP positive	4 (9.3%)
*Skin involvement*	
Active (mRODNAN skin score ≥ 14)	21 (56.8%)
Inactive (mRODNAN skin score < 14)	16 (43.2%)
*Organ involvement: (number/all patients; %)*	
GI*** involvement	25/38 (65.8%)
Lung involvement	33/38 (86.8%)
Heart involvement	15/27 (55.6%)
Diabetic	4 (9.3%)

*Limited cutaneous systemic sclerosis

**Diffuse cutaneous systemic sclerosis

***Gastrointestinal

**Table 2:** Hand Radiological Findings in patients with systemic sclerosis compared with other studies

	Our study	Other studies
Acro-osteolysis	25 (58.2%)	(9%–55%)
Mild	10 (23.3%)	
Moderate	10 (23.3%)	
Severe	5 (11.6%)	
Calcinosis	3 (6.9%)	(10%–37.8%) and 10%
Mild	1 (2.3%)	
Moderate	2 (4.6%)	
Joint space narrowing	24 (55.8%)	(17.9%–75.3%)
Mild	6 (14%)	
Moderate	14 (32.6%)	
Severe	4 (9.3%)	
Marginal erosions	15 (34.9%)	(6.7%–17.1%)
Mild	6 (14%)	
Moderate	8 (18.6%)	
Severe	1 (2.3%)	
Peri articular calcifications	2 (4.7%)	(6.6%–35.7%)
Mild	1 (2.3%)	
Moderate	1 (2.3%)	
Juxta-articular osteoporosis	40 (93%)	(12%–22.6%)
Mild	9 (20.9%)	
Moderate	27 (62.8%)	
Severe	4 (9.3%)	
Flexion deformity	6 (14%)	(7%–55%) and 7%
Moderate	3 (7%)	
Severe	3 (7%)	
Subluxation	5 (11.6%)	(4.8%–23%)
Mild	4 (9.3%)	
Moderate	1 (2.3%)	
Diffuse osteopenia	8 (18.6%)	(9.7%–46%)
Mild	3 (7%)	
Moderate	5 (11.6%)	
Pencil-in-cup deformity (mild)	3 (7%)	1.3%

**Table 3:** Foot Radiological Findings in patients with systemic sclerosis compared with other studies

	Our study	Other studies
Acro-osteolysis	20 (46.5%)	8%
Mild	6 (14%)	
Moderate	12 (27.9%)	
Severe	2 (4.7%)	
Hallux valgus	10 (23.3%)	26.3%
Mild	3 (7%)	
Moderate	7 (16.3%)	
Joint space narrowing	25 (58.1%)	(5%–40%)
Mild	8 (18.6%)	
Moderate	16 (37.2%)	
Severe	1 (2.3%)	
Erosions	7 (16.3%)	(2.6%–13%)
Mild	2 (4.7%)	
Moderate	4 (9.3%)	
Severe	1 (2.3%)	
Para articular calcifications (moderate)	1 (2.3%)	2.6%
Juxta-articular osteoporosis	40 (93%)	6.6%
Mild	15 (34.9%)	
Moderate	24 (55.8%)	
Severe	1 (2.3%)	
Subluxation	19 (44.2%)	(13.1%–16%)
Mild	12 (27.9%)	
Moderate	6 (14%)	
Severe	1 (2.3%)	
Diffuse osteopenia	11 (25.6%)	44.7%
Mild	3 (7%)	
Moderate	8 (18.6%)	
Pencil-in-cup deformity	3 (7%)	1.3%
Mild	1 (2.3%)	
Moderate	2 (4.7%)	
Tarsal degenerative pattern	9 (20.9%)	3.9%
Mild	1 (2.3%)	
Moderate	7 (16.3%)	
Severe	1 (2.3%)	

8 patients had at least one severe radiographic change in the hand or foot. All of them were negative for anti-ccp antibody and except one whose skin type was unspecified; others had active skin involvement.

The presence of anti-ccp antibody was detected in 4 (9.3%), while positive RF was found in 13 (30.2%) of SSc patients. 12 was the lowest value accepted as a positive anti-ccp antibody. 25% of them had cardiac involvement and 75% had lung involvement. Except for one whose GI involvement was unspecified, all the others had GI involvement. All of them had hand joint space narrowing and hand and foot juxta-articular osteoporosis. Other clinical, laboratory and radiological characteristics of patients with SSc and positive anti-ccp antibodies are shown in Table 4.

**Table 4:** Clinical features of 4 female patients with systemic sclerosis and positive anti-ccp antibody

Patient	Age (year)	Disease duration (year)	SSc subset	RF	Anti-CCP anitibody titre positive ≥ 6	Organ involvement				Radiographic abnormalities									
						Heart	Skin	GI	Lung	Hand					Foot				
										Acro-osteolysis	Joint space narrowing	Marginal erosion	Juxta-articular osteoporosis	Subluxation	Joint space narrowing	Juxta articular osteoporosis	Subluxation	Hallux valgus	Periarticular calcification
1	32	8	dSSc	+	67.8	–	Active	+	+	2 +	2 +	2 +	2 +	1 +	–	2 +	2 +	1 +	–
2	50	25	lSSc	–	12.7	–	Inactive	*	+	–	1 +	–	2 +	–	–	2 +	1 +	1 +	2 +
3	66	20	lSSc	+	241	+	Inactive	+	+	–	2 +	2 +	2 +	–	1 +	1 +	–	–	–
4	42	2	dSSc	–	23	–	Active	+	–	–	1 +	1 +	1 +	–	1 +	1 +	–	–	–

+ *1* mild, + *2* moderate

*Unspecified

### Association between radiologic findings and features

#### Hand

The prevalence of joint space narrowing and acro-osteolysis were higher in subjects with active skin involvement (mRSS > 14) [16/21 vs. 4/16 for patients with inactive skin involvement (mRSS < 14); *P* value = 0.002]. Marginal erosion was significantly associated with GI involvement (13/25 vs. 0/13 for patients without GI involvement, *P* value = 0.001). Subluxation or flexion deformity tended to be associated with active skin involvement (mRSS > 14) [5/21 vs. 0/16 in patients with inactive skin involvement (mRSS < 14); *P* value = 0.05]. We found no significant difference between patients with and without other radiological changes in terms of clinical features, autoantibody profile, or organ involvement.

#### Feet

In our study, subluxation was found to be strongly associated with the young age group (< 44 years) (13/21 vs. 6/22 for patients in the middle age group, *P* value = 0.02). Tarsal degenerative pattern showed significant association with long disease duration (> 10 years) [7/19 vs. 2/24 for patients with short disease duration (< 10 years); *P* value = 0.03]. Diffuse osteopenia was associated with non-cardiac involvement (0/15 vs. 4/12 for patients with cardiac-involvement; *P* value = 0.02). It means that from 27 patients for whom cardiac-involvement had been evaluated; only four patients had diffuse osteopenia and none of them had cardiac involvement. Acro-osteolysis tended to be associated with the absence of rheumatoid factor (17/30 patients vs. 3/13 for patients with the presence of rheumatoid factor; *P* value = 0.05). The frequency of digital ulcers in our study population was less than 20% which may explain this lower-level difference. We found no significant difference between patients with and without other radiological changes in terms of clinical features, autoantibody profile, or organ involvement.

## Discussion

Our results highlight the striking level of hand and foot involvement in SSc, as evaluated by X-ray. The severity and frequency of these findings are demonstrated in Tables 2 and 3.

Hand acro-osteolysis was seen in 58.2% of patients. It is nearly in agreement with the prevalence of other reports (9%–55%) [4–7, 12, 13, 17, 23, 32]. The distribution of hand acro-osteolysis severity in our patients was 65.1% (normal or mild) and 34.9% (moderate or severe). They were nearly in agreement with the prevalence of a study in England [10] and higher than another research in Iran [24]. We have found a higher frequency of foot acro-osteolysis (46.5%) than La Montagna's findings (8%) [5].

Prevalence of hand calcinosis in our series (6.9%) is lower than that reported in previous studies (10%–37.8%) [3–7, 9, 12, 13, 16, 17, 23, 24, 33]. But it is nearly in agreement with the recent study of Sakata and coworkers (10%) from Japan in 2019. Due to previous reports, Calcinosis was significantly associated with and most often seen in patients with digital ulcers. Digital ulcers were identified as independent predictors of the radiographic progression of calcinosis [4, 15].

We have found hand marginal erosion in 34.9% of patients, whereas was reported (6.7–17.1%) [7, 11, 16]. For a careful distinction between osteoarthritis- and inflammatory-related erosions Koutaissoff and coworkers [7] introduce marginal erosion. Marginal erosions are typical of arthritis while surface erosions are mostly observed in spondyloarthropathy or Calcium pyrophosphate dihydrate crystal deposition (CPPD) disease. We think this difference is because they grouped normal (score 0) and doubtful lesions (score 1) together (the corresponding areas were considered as “normal”), and evident (score 2) and severe lesions (score 3) together (the corresponding areas were considered as “abnormal”). But we grouped normal (score 0) and abnormal (score 1, 2, 3). Foot erosion was seen in 16.3% of our study group and is nearly in agreement with the prevalence of other reports (2.6%–13%) [3, 5, 21, 26].

The frequency of hand juxta-articular osteoporosis in SSc had been estimated at 12%–22.6% in previous studies [3, 7, 9, 11] and was 93% in our series. The frequency of foot juxta-articular osteoporosis in SSc had been estimated at 6.6% in previous studies [3] and was 93% in our series. Due to Koutaissoff and coworkers’ findings in their case–control study of 167 patients and 168 of age- and gender-matched controls; juxta-articular osteoporosis had never been observed in controls [7]. In Thietart et al. [34] case–control cohort study; they found that SSc may be a risk factor for low bone mineral density (BMD), with no significant association with the usual osteoporosis risk factors. Unless previous studies which the mean age was 52 and most of their patients were post-menopausal women, the mean age of our patients was 44.79 and only 35% were females around the age of 50. Overall, we could rule out the possibility of such an arthropathy, unrelated to SSc, occurring in our patients and we think it may represent a specific arthropathy in scleroderma.

The frequency of hand flexion deformity was 14% and agrees with previous studies (7%–55%) [3–6, 9, 16, 23]. It is close to Sakata and coworkers [23] published data (7%) from Japan in 2019. They said that flexion contracture was more prevalent in SSc patients with digital tip ulcers or digital pitting scars.

Hand subluxation was seen in 11.6% of our patients and agrees with the prevalence of previous reports (4.8%–23%) [3, 5, 9, 23, 32]. Frequency of foot subluxation in our study group was higher (44.2%) than in previous studies (13.1%–16%) [3, 5]. Weight-bearing could be a risk factor but interestingly we have found a significant association between foot subluxation and the young age group (*P* value = 0.02). This is a new relation; So, we hypothesized that this may be due to specific arthropathy in SSc patients.

Prevalence of hand joint space narrowing was 55.8% and agrees with the prevalence of other reports (17.9%–75.3%) [4–7, 9, 15, 17, 21, 23, 32]. We have found foot joint space narrowing in 58.1% of patients but previous reports do not support this finding (5%–40%) [5, 21]. Foot para-articular calcification was rare in our series of SSc patients (2.3%) consistent with frequency reported in other series (2.6%) [3]. The frequency of hand diffuse osteopenia was 18.6% and agrees with the prevalence of other reports (9.7%–46%) [3, 9]. The frequency of foot diffuse osteopenia was lower (25.6%) than in La Montagna and coworkers’ study (44.7%) [3]. Hand periarticular calcification was demonstrated in 6.6%–35.7% of previously reported SSc patients [3, 7] and was 4.7% in our study population. Hand or foot pencil-in-cup deformity was seen in 7% of patients. It is nearly in agreement with the prevalence of other reports (1.3%) [3]. Foot hallux valgus was seen in 23.3% of patients. It is nearly in agreement with the prevalence of other reports (26.3%) [3]. The frequency of foot tarsal degenerative pattern in our study group was higher (20.9%) than in previous studies (3.9%) [3].

These very disparate results may be accounted for differences in the number of patients, the combination of retrospective and prospective data, characteristics of the study population, the role of longitudinal studies on the hypothesis that foot changes began later, and few studies especially on foot involvements. It is worth mentioning that most of the radiological involvements we found; had a higher prevalence than the previous studies.

Foot tarsal Degenerative Pattern was found to be significantly associated with long disease duration (> 10 years) (*p* = 0.03). There isn’t any other study to assess this relation. We hypothesized that degenerative changes could happen over time.

Foot diffuse osteopenia was associated with non-cardiac involvement (*P* value = 0.02). It means that of those who had cardiac involvement, none of them had diffuse osteopenia. Up to now, there isn’t any study that has exactly assessed this relation. Ashida and coworkers reported a significantly greater prevalence of heart involvement in the patients with Contracture of phalanges [35]. Tas and coworkers in 2012 reported a correlation between heart involvement and arthritis (concomitant erosions and joint space narrowing) [6].

We have found a significant association between hand marginal erosion and GI involvement (*P* value = 0.001). Ashida and coworkers reported significantly greater prevalence of esophageal involvement in the patients with Contracture of phalanges [35]. Erre and coworkers found a significantly association between finger flexion deformities or bone resorptions and esophageal involvement. Something that highlights the role of the fibrotic process on disease expression [9]. Sakata and coworkers found an association between calcinosis and GI involvement and between flexion contracture and GI involvement [23]. Due to Koutaissoff and coworkers’ findings in their case–control study of 167 patients and 168 of age- and gender-matched controls; Marginal erosions had never been observed in controls [7]. Keeping in mind this definition, and the previous reported association of bone resorption and esophageal involvement is consistent with the existence of a specific arthropathy in SSc unexplained by other arthropathies.

We have found a significant association between hand joint space narrowing or hand acro-osteolysis and active skin involvement (mRSS > 14) (*P* value = 0.002). Hand subluxation or hand flexion deformity tended to be associated with active skin involvement (mRSS > 14) (*P* value = 0.05). Due to Koutaissoff and coworkers [7] patients with tuft calcinosis had a significantly higher Rodnan-modified total skin score. La Montagna and coworkers found a significant correlation between flexion contractures in the hands and the severity score of skin (mRSS) [3]. It cannot be excluded that cutaneous and subcutaneous sclerosis or tenderness and/or swelling of joints have a determinant role in conditioning the joint anatomy. Nevertheless, this pathological link that we have found is noticeable. Especially in the case of the fact that previous studies had investigated mean modified Rodnan skin score instead of classifying mRSS. Thus, we think it could reflect a specific arthropathy in SSc unexplained by other arthropathies.

In our survey, positive RF was found in 30.2% of patients and was the same as what was detected in previous studies (3.9%–44%) [3–6, 9, 11, 15, 16]. Acro-osteolysis tended to be associated with the absence of rheumatoid factor (*P* value = 0.05). This is a new association. The presence of anti-ccp antibody was in 9.3% as suggested by published studies (1%–15%) [11, 36–43], so our data support these findings. We have found both joint space narrowing and marginal erosion in 3 out of 4 patients with positive anti-ccp antibody, consistent with the existence of primary erosive arthropathy in SSc unexplained by overlap with rheumatoid arthritis, as suggested by Avouac et al. and Cuomo et al. [4, 11, 17].

Our study has several limitations. It was a cross-sectional and not a longitudinal radiological study, so it prevented the evaluation of the natural history of the damage in SSc. It was a retrospective study and analyzed a small number of patients. Large studies are required to confirm our results. Other limitations were the observational character of the trial and the lack of a control group. Also, juxta-articular osteoporosis is a subjective finding and it may have caused overdiagnosis in our study.

## Conclusion

In conclusion, this study confirms that arthropathy is prevalent in SSc patients. Juxta-articular osteoporosis is the most common radiological manifestation of systemic sclerosis arthropathy. There seems to be a significant relationship between radiological hand and foot involvement and active skin involvement, which requires studies with a larger sample size. Radiological involvements necessitate conducting studies with a larger sample size and considering a healthy control group.

## Data Availability

Data supporting the findings of this study are available from the corresponding author, but restrictions apply to the availability of these data, which were used under license for the current study, and are therefore not publicly available. However, the data is available in the form of an Excel file from the authors upon reasonable request and with the permission of Nafiseh Abdolahi.

## Abbreviations

SSc: Systemic sclerosis
FBS: Fasting blood glucose
HbA1c: Hemoglobin A1c
RF: Rheumatoid factor
Anti-CCP: Anti-cyclic citrullinated peptide
LEVF: Left ventricular ejection fraction
PAP: Pulmonary arterial pressure
FVC: Forced vital capacity
PFT: Pulmonary function tests
PAH: Pulmonary arterial hypertension
UCLA SCTS: University of California at Los Angeles scleroderma clinical trial consortium
CT: Computed tomography
LcSSc: Limited cutaneous systemic sclerosis
DcSSc: Diffuse cutaneous systemic sclerosis
CPPD: Calcium pyrophosphate dihydrate crystal deposition
BMD: Bone mineral density
